# The prevalence of dental fluorosis and exposure to fluoride in drinking water: A systematic review

**DOI:** 10.15171/joddd.2016.021

**Published:** 2016-08-17

**Authors:** Fatemeh Goodarzi, Amir Hossein Mahvi, Mostafa Hosseini, Saharnaz Nedjat, Ramin Nabizadeh Nodehi, Mohammad Javad Kharazifard, Mina Parvizishad, Zahra Cheraghi

**Affiliations:** ^1^Msc in Environmental Health Engineering, Department of Environmental Health Engineering, School of Public Health, Tehran University of Medical Sciences, Tehran, Iran; ^2^Asistant Professor, Department of Environmental Health Engineering, School of Public Health, Tehran University of Medical Sciences, Tehran, Iran; ^3^Center for Solid Waste Research, Institute for Environmental Research (IER), Tehran University of Medical Sciences, Tehran, Iran; ^4^Professor, Department of Epidemiology and Biostatistics, School of Public Health, Tehran University of Medical Sciences, Tehran, Iran; ^5^Professor, Department of Environmental Health Engineering, School of Public Health, Tehran University of Medical Sciences, Tehran, Iran; ^6^DDS, PhD, Dental Research Center, Dentistry Research Institute, Tehran University of Medical Sciences, Tehran, Iran; ^7^Msc in Environmental Health Engineering, Department of Environmental Health Engineering, School of Public Health, Tehran University of Medical; ^8^PhD Student in Epidemiology, Department of Epidemiology and Biostatistics, School of Public Health, Tehran University of Medical Sciences, Tehran, Iran

**Keywords:** Dental fluorosis, fluorides, systematic review, water

## Abstract

***Background.*** Regarding the lack of comprehensive systematic review on the efficacy of water fluoridation and prevalence of dental fluorosis, the aim of the current research was to systematically study the prevalence of dental fluorosis at different levels of water fluoride in the world and lay emphasis on the amount of fluoride in drinking water.

***Methods.*** Studies were searched in PubMed, Scopus, SID, and IranMedex, with regard to inclusion criteria. Study validity was assessed with some checklists, and analyses were performed to ascertain the prevalence of dental fluorosis among individuals categorized in age groups.

***Results.*** Investigation of the heterogeneity and analysis of the subgroups revealed that in the 6-18 year age group, when water fluoride level was less than 0.7 ppm and there was exposure to water fluoride in the first 6-8 years of life, no significant heterogeneity was detected among the studies in this subgroup. Thus, the pooled estimation of dental fluorosis prevalence in this subgroup was 12.9% (95% CI: 7.5-18.3%). Furthermore, meta-regression indicated that the exposure time to fluoride in drinking water, or exposure to fluoride in supplements, diets, air, etc as well as the quality of studies had a significant relation to the difference in the prevalence of dental fluorosis.

***Conclusion.*** The results revealed no heterogeneity in just 2 subgroups, and the results of subgroups could be pooled in them. Furthermore, the number of studies included in this review considerably decreased by considering all the detected confounding factors, whereas other similar systematic reviews mentioned at most 2 factors.

## Introduction

Water fluoridation is considered as an effective method to prevent dental problems and was first applied in 1945 in some regions in the United States.^1^ The initial epidemiologic surveys on water fluoridation and its relation to dental problems were carried out by Dean et al^2,3^ in 1941 and 1942. Also, a series of epidemiologic surveys were performed in areas with natural fluoridation in Japan. Although these surveys paved the way for epidemiologic studies of water fluoridation and dental fluorosis, many of them did not assess the association between the fluoride level of drinking water and dental fluorosis.^4-6^ However; such association has been confirmed by most of the researches since surveys carried out by Dean et al.^7-11^

Few surveys into the relationship between the amount of fluoride in drinking water and dental fluorosis have been carried out in East African countries.^12,13^ Although data regarding the amount of fluoride in water and percentage of dental fluorosis from West Africa is limited, the prevalence of dental fluorosis in 12‒15-year-old children was examined in relation to the level of fluoride in drinking water through a comprehensive research in central Nigeria.^14^ In another study in Norway, dental fluorosis was identified as a main cause for consumption of fluoride supplements. The participants in this survey used high-dose supplements of fluoride since 1987.^15^ Furthermore, a recent study was conducted in the central region of Mexico for identifying the impact of under-nutrition on dental fluorosis. In this research, 734 school children were chosen from 3 districts with different fluoride levels of drinking water. The results indicated that children who had low height-for-age (as a consequence of inadequate nutrition) were at a higher risk for dental fluorosis.^16^

Recently, numerous studies have investigated the effect of fluoride on the prevalence of dental fluorosis. These studies should be reviewed systematically. In this regard, the main objective of this review was to provide an overview of studies with regard to a given research question, and the first step was to conduct a comprehensive review of the relevant literature. The initial review of relevant studies revealed that three systematic reviews have been conducted on the prevalence of dental fluorosis, and exposure to fluoride in drinking water. McDonagh et al^17^ carried out a systematic review about water fluoridation in 2000. In this study, 214 published articles were reviewed without any limitations in time and place, and the subject of 88 out of 214 articles was dental fluorosis. The findings of this study revealed a significant relationship between the prevalence of dental fluorosis and the amount of fluoride in drinking water. This study was carried out about 15 years ago, and the results are not up-to-date. Thus, further research was needed in order to consider new articles in this field.^17^

Another systematic review was conducted by Azami-Aghdash et al,^18^ which considered the prevalence of dental fluorosis and fluoride level in drinking water separately. This review was limited to some regions in Iran, and its findings showed that despite the low level of fluoride in water, the prevalence of dental fluorosis was high. Also, the average amount of fluoride in drinking water and the average prevalence of fluorosis were measured separately.^18^

Australian National Health and Medical Research conducted a systematic review in Australia on the efficacy and safety of water fluoridation. In this study just the articles published in English between 2000 and 2007 were added^19^ to the articles of McDonagh et al in 2000.^17^ Furthermore, the same method‏ was applied, and the findings showed a negative correlation between water fluoridation and the frequency of dental caries.^19^

The most important shortcoming of prior systematic reviews on the fluoride level of drinking water and prevalence of dental fluorosis was a lack of comprehensive analyses. Therefore, a comprehensive study was necessary. In this regard, the current systematic review focused on the fluoride level of drinking water and prevalence of dental fluorosis in all the age groups. Thus, the exposure time to fluoride in drinking water and exposure to fluoride were considered as confounding factors, and the outcomes were presented in terms of the prevalence of dental fluorosis.

## Methods

### Search strategy

The databases which were used in this research included PubMed (http://www.ncbi.nlm.nih.gov/pubmed), Scopus (http://www.scopus.com/), SID (http://sid.ir/), and IranMedex (http://www.iranmedex.ir/). The time scope covered the starting date of the database to December 2014. Also, further searches were conducted through bibliographies of the included studies and also previous systematic reviews, especially that of McDonagh et al,^17^ Index Medicus, and Excerpta Medica. All studies were included irrespective of the language.^17^

### Inclusion criteria 

There was no protocol for the current systematic review, and the studies that met the following inclusion criteria were eligible for abstraction:

- Being a primary study (not a review study).
- Studying the human beings.
- Being related directly to fluoride in drinking water supplies and dental fluorosis.
- At least one group of individuals having been studied.
- Reporting the measurable outcomes (i.e. Dean’s index) in a group in association with the amount of fluoride in drinking water supply.

### Data acquisition and assessment of the study validity

At least 2 reviewers assessed the inclusion criteria, extracted data from studies and examined the validity independently. Then, the third reviewer checked the results. Also, any disagreement among reviewers was resolved through consensus. It should be pointed out that they were not blinded to the names of the authors and other information of the articles. Considering the Kappa coefficient, the inter-observer reliability was 85%. The study validity was assessed in three ways using the STROBE checklist modified for cross-sectional study designs and the checklist of Centre for Evidence-based Social Service modified for pre-post study designs in this study. Each item of the checklist was assigned 1 point, and a complete score was allocated to high-quality articles. Furthermore, scores of 0.5 and 0 were allocated, respectively, to moderate-quality and low-quality items. Finally, the scores of all the items were gathered, and the overall quality level of the study was determined based on the attitude of the reviewer. Subsequently, the quality of the article was reported as low, moderate and high.

A more specialized validity assessment was carried out about the method of determining the Dean’s index for dental fluorosis. The checklists for these two validity assessments were developed by the Community Oral Health group. A score was given to each item, and then the articles were grouped in three categories of low-, moderate- and high-quality with regard to the total score:.

- The overall score in examining the dental fluoro-sis (4 for high quality, 2‒3.99 for medium quality, and <2 for low quality was).
- The overall score in determining the fluoride level of drinking water supplies (10‒13 for high quality, 6‒9.99 for medium quality, and <6 for low quality).

Finally, level A (high or moderate quality) or level B (low quality) was given regarding the results of the assessments.

### Outcome measures

Dean’s index was used to classify dental fluorosis. Dental fluorosis was considered in this review as any level other than normal and questionable level on Dean’s index. Furthermore, meta-analysis was conducted for obtaining the summary measure with 95% confidence interval (CI). Stata /SE 11.1 (Stata Corp LP, College Station, TX 77845, USA) was employed for data analysis, and random effect models were used for reporting the results.^20^

### Heterogeneity

The heterogeneity was examined using the chi-squared test at 10% significance level. Also, inconsistency was quantified across studies using І^2^ statistic,^21^ and the difference between study variance was estimated using τ^2^ statistics.^22^

### Analysis

Effect size was plotted (95% confidence intervals) when data were in right format. Also, heterogeneity was assessed by examination of graphs, and employing the‏ χ^2^ statistics. Furthermore, meta-regression was conducted in case of significant inconsistency, and random effects models were used for combining the results. Stata/SE 11.1 (StataCorp LP, College Station, TX 77845, USA) was used for analysis.

Data categorization and analysis of subgroups were conducted to eliminate the effect of confounding factors. Regarding the dental‏ fluorosis, categorization was first performed based on age. According to dentists and epidemiologists, age is the most important variable in dental fluorosis. The second most important variable was the amount of fluoride in drinking water. Hence, the categorization in the next step was based on fluoride level in drinking water. Exposure time to fluoride in drinking water and any exposure to fluoride in supplements, diet, air, etc can also affect the prevalence of dental fluorosis, which was categorized at the third place based on one of them. The categorization of variables was performed as follows:

- Age (year): Under 6, 6‒18, and over 18.
- The amount of fluoride in drinking water (PPM): Under 0.7, 0.7‒1.2, 1.3 to 2, and over 2.
- Any exposure to fluoride in supplements, diet, air, etc: Yes, no, and not reported.
- Exposure time to fluoride in drinking water: Lifetime exposure, exposure from 6 to 8 years in early life, and not reported.

In the next step, a categorization was conducted based on age, fluoride level of drinking water, and quality of studies. These three categorizations were carried out just for 6‒18-year age group because the two other age groups were excluded from this review for the reasons mentioned above. Finally, 3 groups were identified. Each group was categorized in subgroups to illustrate the prevalence of dental fluorosis with regard to these variables.

### Study characteristics

In the current review, all the age groups were considered for analysis, and confounding factors included the exposure time to fluoride in drinking water, as well as the exposure to fluoride in supplements, diet, air, etc. Comparison of dental fluorosis prevalence was made with the groups having been exposed to different levels of fluoride in drinking water. Also, pre-post, cross-sectional, ecological and cohort study designs were considered.

It should be noted that the risk of bias for the prevalence of fluorosis across studies was unjustifiable, and there was no assumption of bias accordingly.

## Results

### Including articles

The steps in which 57 out of 2402 articles were included are explained here:

 1) Extraction of 2402 articles from databases (1125 articles from Scopus, 1032 articles from PubMed, 115 articles from IranMedex, and 130 articles from SID).

 2) A total of 1212 articles were excluded because of duplication (1190 articles remained).

 3) A total of 1082 articles were excluded because they did not meet the inclusion criteria (108 articles remained).

 4) A total of 51 articles were not considered in the analysis because of the following reasons:

- The fluoride level in drinking water was over 6 ppm.
- The amount of fluoride in drinking water was measured, but it was not mentioned in the article.
- There was a wide range of fluoride level in drinking water.
- The individuals did not have sufficient exposure to fluoride-containing water.
- In the studies, the amount of fluoride in drinking water could not be categorized based on the groups defined for fluoride level in drinking water.
- Being an endemic area for the prevalence of dental fluorosis within the scope of the study.
- The individuals could not be categorized based on the groups defined for ages.
- The prevalence of the dental fluorosis was mentioned for all the age groups.
- The age of individuals was not mentioned.
- Two diverse age groups of 7 and 20 years were categorized in one age group.
- The prevalence of the dental fluorosis had not been mentioned based on the fluoride level.
- Measures of the prevalence of dental fluorosis were presented graphically. The exact data could not be extracted from the graphs.
- The dental fluorosis was reported based on CFI.
- At first, the region with high prevalence had been determined, and then the amount of fluoride had been measured.
- Considering prior studies, the results were duplicated in some cases.
- It was not mentioned whether the prevalence of dental fluorosis included the questionable level on Dean`s index or not.
- The prevalence of dental fluorosis included the questionable to severe level.

5) Finally, 57 articles (152 study areas) containing all the age groups were considered for analysis, and the studies in which the average age of the study population was under 6 years (1 article, 1 study area) or over 18 years (4 articles, 6 study areas) were excluded. Since there were not enough articles in these age groups to provide data for analysis, 52 articles included 145 study areas, in which the average age of individuals was between 6 and 18 years. In the majority of dental fluorosis studies, more than one study area was assessed.

### Determining the quality of studies

All the studies were cross-sectional, except one that was pre-post trail. A total of 42 articles (111 study areas) were of evidence level A (high or moderate quality), and 15 out of these articles (41 study areas) were of evidence level B (low quality). 

### Examining the heterogeneity of studies

As it was mentioned in the “Materials and Methods”, the groups were categorized in subgroups based on the amount of fluoride in drinking water, the exposure time to fluoride in drinking water, and any exposure to fluoride in supplements, diet, air, etc, or the quality of studies for the 6‒18-years age group. Group 1 and group 2 were categorized in 12 subgroups, and group 3 was categorized in 8 subgroups.

According to Table 1, group 1 was categorized into subgroups based on the amount of fluoride in drinking water and the exposure time for the 6‒18-year age group. In this group, the heterogeneity was significant (P < 0.001) among researches in all the subgroups except for subgroup 2. Thus, just the result of the studies in the subgroup 2 could be combined. However, in the same level of water fluoride, the prevalence of dental fluorosis in subgroups with lifetime exposure was more than that in subgroups with fluoride exposure in the first 6‒8 years of life. It should be noted that studies in which the exposure time to water fluoride was not reported were not considered in this review.

**Table 1:** Categorizing group 1 into subgroups based on fluoride level in drinking water and the exposure time in the 6‒18-year age group

**Subgroup number**	**Fluoride level (ppm)**	**Exposure time**	**Number of studies**	**I** ^2*^	***P*** -Value^**^	Pooled estimation of dental fluorosis prevalence (CI^***^)
**1**	< 0.7	Lifetime	38	99.9%	< 0.001	23.6% (14.5%, 32.8%)
**2**	< 0.7	First 6-8 years of life	3	0%	0.397	12.9% (7.5%, 18.3%)
**3**	< 0.7	Not reported	18	-	-	-
**4**	0.7-1.2	Lifetime	24	99.5%	< 0.001	41.7% (29.9%, 53.5%)
**5**	0.7-1.2	First 6-8 years of life	1	-	-	18.5% (12.4%-24.6%)
**6**	0.7-1.2	Not reported	8	-	-	-
**7**	1.3-2	Lifetime	9	99.3%	< 0.001	65.9% (46.8%, 85.0%)
**8**	1.3-2	First 6-8 years of life	1	-	-	39.7% (27.0%, 52.4%)
**9**	1.3-2	Lifetime	5	-	-	-
**10**	> 2	Lifetime	18	98.8%	< 0.001	77.0% (67.2%, 86.7%)
**11**	> 2	First 6-8 years of life	1	-	-	29.4% (16.9%, 41.9%)
**12**	> 2	Not reported	19	-	-	-

^*^ I-squared (variation in estimation attributable to heterogeneity) ^* *^ P-Value (based on heterogeneity statistics) ^***^CI: Confidence Interval

As it is illustrated in Table 2, group 2 was categorized into subgroups considering the amount of fluoride in drinking water and any exposure to fluoride in supplements, diet, air, etc for the 6‒18-year age group. Significant heterogeneity (P < 0.001) was found among the studies in all the subgroups except for subgroup 11. Hence, the results of the studies in subgroup 11 could be pooled but others could not. The studies in which exposure to fluoride in supplements, diet, air, etc was not reported, were not considered in this review.

**Table 2:** Categorizing group 2 into subgroups based on fluoride level in drinking water and any exposure to fluoride in supplements, diet, air, etc. for the 6‒18-year age group

**Subgroup number**	**Fluoride level (ppm)**	**exposure to fluoride in supplements, diet, air, etc.**	**Number of studies**	**I** ^2*^	**P-Value** ^**^	Pooled estimation of dental fluorosis prevalence (CI^***^)
**1**	< 0.7	Yes	27	94.4%	< 0.001	16.0% (12.6%, 19.5%)
**2**	< 0.7	No	7	97.9%	< 0.001	24.9% (12.4%, 37.3%)
**3**	< 0.7	Not reported	25	-	-	-
**4**	0.7-1.2	Yes	15	99.8%	< 0.001	37.1% (21.9%, 52.3%)
**5**	0.7-1.2	No	2	78.5%	0.031	16.3% (11.6%, 21.0%)
**6**	0.7-1.2	Not reported	16	-	-	-
**7**	1.3-2	Yes	5	96%	< 0.001	33.7% (16.3%, 51.0%)
**8**	1.3-2	No	0	-	-	-
**9**	1.3-2	Not reported	10	-	-	-
**10**	> 2	Yes	5	98.9%	< 0.001	73.2% (54.6%, 91.8%)
**11**	> 2	No	3	28.8%	0.246	98.3% (96.4%, 100%)
**12**	> 2	Not reported	30	-	-	-

^*^ I-squared (variation in estimation attributable to heterogeneity) ^**^ P-Value (based on heterogeneity statistics) ^***^CI: Confidence Interval

According to Table 3, group 3 was categorized into subgroups based on fluoride level in drinking water and quality of studies for the 6‒18-year age group. Significant heterogeneity (P < 0.001) was found among the studies in all the subgroups. Thus, the results of the studies in all subgroups could not be pooled.

**Table 3:** Categorizing group 3 into subgroups based on fluoride level in drinking water and quality of studies for the 6‒18-year age group

**Subgroup number**	**Fluoride level (ppm)**	**Quality of studies**	**Number of studies**	**I** ^2*^	**P-Value** ^**^	Pooled estimation of dental fluorosis prevalence (CI^***^)
**1**	< 0.7	level A	59	99.9%	< 0.001	17.8% (13.0%, 22.5%)
**2**	< 0.7	level B	0	-	-	-
**3**	0.7-1.2	level A	21	99.5%	< 0.001	29.8% (53.5%, 41.0%)
**4**	0.7-1.2	level B	12	99.4%	< 0.001	25.5% (15.5%, 35.5%)
**5**	1.3-2	level A	9	99.0%	< 0.001	38.4% (16.8%, 59.9%)
**6**	1.3-2	level B	6	96.2%	< 0.001	83.4% (75.3%, 91.5%)
**7**	> 2	level A	31	98.3%	< 0.001	75.6% (68.5%, 82.6%)
**8**	> 2	level B	7	98.6%	lt; 0.001	73.5% (53.3%, 93.6%)

^*^ I-squared (variation in estimation attributable to heterogeneity) ^* *^ P-Value (based on heterogeneity statistics) ^***^CI: Confidence Interval

**Figure 1.**
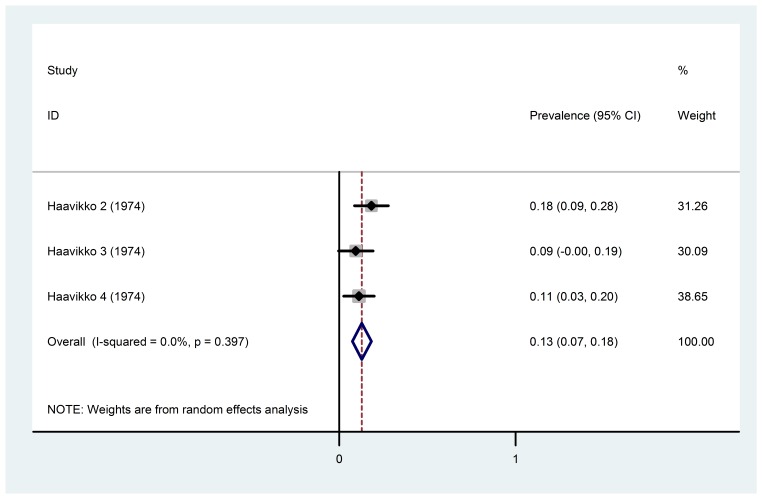
Forest plot of the prevalence of dental fluorosis in the 6‒18-year age group with fluoride level in drinking water under 0.7 ppm, and exposure time to fluoride in drinking water in the first 6‒8 years of life (subgroup 2).

Regarding the forest plot in Figure 1, in the 6‒18-year age group, the amount of fluoride in water (under 0.7 ppm) and fluoride exposure time in the first 6‒8 years of life (subgroup 2), there was no heterogeneity, and the pooled estimate of the prevalence of dental fluorosis could be reported. It can be noted that the prevalence of dental fluorosis in this subgroup was 12.9% (95% CI: 7.5‒18.3%).

**Figure 2.**
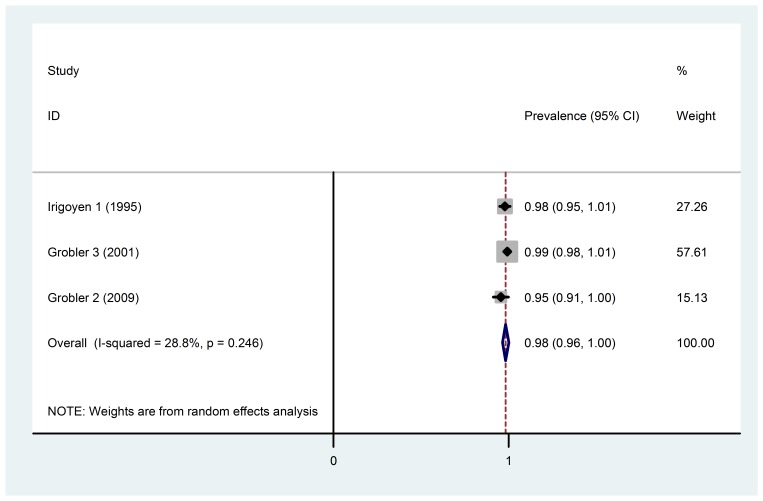
Forest plot of the prevalence of dental fluorosis in the 6‒18-year age group with fluoride level in drinking water over 2 ppm, and individuals without any exposure to fluoride in supplements, diet, air, etc (subgroup 11).

Regarding the forest plot in Figure 2, in the 6‒18-year age group, the amount of fluoride in drinking water (over 2 ppm) and individuals without any exposure to fluoride in supplements, diet, air, etc. (subgroup 11), there was no heterogeneity. Hence, the results of the prevalence of dental fluorosis could be pooled. The prevalence in this subgroup was 98.3% (95% CI: 96.4‒100%).

### Meta-regression

Meta-regression showed that the exposure time to fluoride in drinking water, or any exposure to fluoride in supplements, diets, air, etc, as well as the quality of studies, had a significant relation to the difference in the prevalence of dental fluorosis. These variables were a source of heterogeneity. Hence, the studies could be grouped, and the analysis could be conducted in the groups. The results of meta-regression are given in Table 4.

**Table 4:** The results of meta-regression for identifying the source of heterogeneity in studies*

**Independent variable**	**Coefficient**	**SE** ^**^	**t**	**P > | t |**	[95 % CI^***^]
**Age > 18 yr**	0.005	0.174	0.030	0.978	-0.340, 0.350
**Water fluoride level**	>0.010	0.009	1.110	0.269	-0.008, 0.027
**Age, 6-18 years**					Reference
**Exposure during the first 6-18 years of life**	-0.174	0.125	-1.400	0.165	-0.421, 0.073
**No report of exposure time**	-0.103	0.065	-1.570	0.119	-0.232, 0.027
**Life-time exposure**					Reference
**Exposure to fluoride in supplements, diet, air, etc.**	-0.182	0.063	-2.880	0.005	-0.307, 0.057
**No exposure to fluoride in supplements, diet, air, etc.**	-0.065	0.116	-0.560	0.579	-0.295, 0.165
**No report on having any exposure to fluoride in supplements, diet, air, etc.**					Reference
**Quality score**	0.020	0.012	1.610	0.110	-0.005, 0.044
**Constant**	0.385	0.092	4.180	0.000	-0.203, 0.568

^*^Number of observations = 134; τ^2^ = 0.099; І^2^ = 99.87%; Adjusted R^2^ = 7.16%; Model F (7,126) =2.44; P > F = 0.022. ^**^Standard Error ^***^CI: Confidence Interval

## Discussion

The main objective of this research was to conduct a systematic review on fluoride level of drinking water and the prevalence of dental fluorosis. The pooled estimation of dental fluorosis prevalence was determined after conducting a meta-analysis. It should be pointed out that the effect of other variables on the prevalence of dental‏ fluorosis was examined. These variables included water fluoride exposure time and any exposure to fluoride in supplements, diet, air, etc. The temperature was also considered as a confounding factor, which has been included in a few studies. Consequently, this factor was not considered in the analysis, and the categorization could not be performed based on it.

Investigation of the heterogeneity and analysis of the subgroups revealed that in the 6‒18-year age group with water fluoride level of less than 0.7 ppm and the water fluoride exposure time in the first 6‒8 years of life, no significant heterogeneity was detected among the studies in this subgroup. The pooled estimation of dental fluorosis prevalence in this subgroup was 12.9% (95% CI: 7.5‒18.3%). Investigation of the heterogeneity and analysis of the subgroups also indicated that in the 6‒18-year age group with water fluoride level of over 2 ppm and without any exposure to fluoride there was no significant heterogeneity among the studies in this subgroup. Furthermore, the pooled estimate of dental fluorosis prevalence in this subgroup was 98.3% (95% CI: 96.4‒100%). However, the results of other studies could not be pooled because of significant heterogeneity in these subgroups.

Considering the above-mentioned facts, the grouping should be performed based on all the confounding factors. In this regard, the following factors were considered:

- Exposure time to water fluoride.
- Exposure to fluoride in supplements, diet, air, etc.
- Water fluoride level in drinking water.
- Quality of study.
- Age.

A closer look at studies revealed that the exposure time to water fluoride, or the exposure to fluoride in supplements, diet, air, etc (as important factors), was not reported in a number of them. Thus, these studies were not included in the analysis, and this limitation reduced the number of pooled studies. Furthermore, we examined the prevalence of dental fluorosis based on all the confounding factors that considerably decreased the number of included studies.

Most of the previous systematic reviews mentioned at most 2 of the above confounding factors. Thus, the significance of heterogeneity in most of the subgroups was mainly due to not considering all the confounding factors. The limitations of similar systematic reviews are:

- The research conducted by McDonagh et al^17^ in 2000 is similar to the current research in terms of scope. However, this review was performed about 15 years ago. Hence, published articles from 2000 to 2015 were considered. The scope of the other similar researches was limited to regions in Iran and Australia.
- None of previous reviews have considered all the above-mentioned confounding factors for grouping and analysis.
- In the research of Azami-Aghdash et al,^18^ fluoride and fluorosis were studied separately.
- In the review which was conducted by Australian National Health and Medical Research, just the English articles were considered for review.^19^

## Conclusion

In this study, the systematic review was conducted through a search in electronic databases, including PubMed, Scopus, IranMedex, and SID. The results of this study were presented as an overview on previous researches on the exposure to fluoride in drinking water and frequency of dental fluorosis. The findings revealed that heterogeneity was not found in only 2 subgroups. Furthermore, the results of these subgroups could be pooled. As one important finding of this study, we can note that most of the prior studies had just mentioned 1 or 2 confounding factors, and consequently, the number of studies included in this research considerably decreased because of considering all the detected confounders.

The implications for further research would be an in-depth investigation on the geographical expansion of dental fluorosis prevalence in different regions considering all the confounding factors to provide a wide epidemiological outlook on different aspects of this issue.

## Acknowledgments

The authors appreciate the financial support of Tehran University of Medical Sciences. Furthermore, authors herewith acknowledge the Department of Community Oral Health in Tehran University of Medical Sciences for affording specialized validity assessment checklist.

## Authors’ contributions

All the authors participated in carrying out this study and supervised writing this article‏. AHM, MH, SN, RN, MJK, and FG designed the study. FG and MP performed the literature review and searched in databases. Furthermore, MH, SN, FG, and ZC performed data analysis. All the authors have read and approved the final manuscript.

## Funding

This study was supported by Tehran University of Medical Sciences.

## Competing interests

The authors declare no competing interests with regards to the authorship and/or publication of this article.

## Ethics approval

Not applicable.
